# Investigating Nonspecific Effects of the Live-Attenuated Japanese Encephalitis Vaccine on Lower Respiratory Tract Infections in Children Aged 25-35 Months: Retrospective Cohort Study

**DOI:** 10.2196/53040

**Published:** 2024-03-18

**Authors:** Siyi Zhan, Hongbo Lin, Yingying Yang, Tao Chen, Sheng Mao, Chuanxi Fu

**Affiliations:** 1 The Institute of Infectious Disease and Vaccine School of Public Health Zhejiang Chinese Medical University Hangzhou China; 2 Center for Disease Control and Prevention of Yinzhou District Ningbo China

**Keywords:** nonspecific effect of vaccines, Japanese encephalitis vaccine, lower respiratory tract infectious diseases, trained immunity, Anderson Gill model

## Abstract

**Background:**

Live attenuated vaccines may be used to prevent nontargeted diseases such as lower respiratory tract infections (LRTIs) due to their nonspecific effects (NSEs).

**Objective:**

We aimed to analyze the NSEs of the Japanese encephalitis vaccine on pediatric LRTIs in children aged 25 months to 35 months.

**Methods:**

A retrospective cohort study was conducted by using a population-based electronic health record database in Zhejiang, China. Enrolled participants were children born from January 1, 2017, to December 31, 2017, and who were inoculated with the live-attenuated Japanese encephalitis vaccine (JE-L) or inactivated Japanese encephalitis vaccine (JE-I) as the most recent vaccine at 24 months of age. The study was carried out between January 1, 2019, and December 31, 2019. All inpatient and outpatient hospital visits for LRTIs among children aged 25 months to 35 months were recorded. The Andersen-Gill model was used to assess the NSEs of JE-L against LRTIs in children and compared with those of JE-I as the most recent vaccine.

**Results:**

A total of 810 children born in 2017 were enrolled, of whom 585 received JE-L (JE-L cohort) and 225 received JE-I (JE-I cohort) as their last vaccine. The JE-L cohort showed a reduced risk of LRTIs (adjusted hazard ratio [aHR] 0.537, 95% CI 0.416-0.693), including pneumonia (aHR 0.501, 95% CI 0.393-0.638) and acute bronchitis (aHR 0.525, 95% CI 0.396-0.698) at 25 months to 35 months of age. The NSEs provided by JE-L were especially pronounced in female children (aHR 0.305, 95% CI 0.198-0.469) and children without chronic diseases (aHR 0.553, 95% CI 0.420-0.729), without siblings (aHR 0.361, 95% CI 0.255-0.511), with more than 30 inpatient and outpatient hospital visits prior to 24 months of age (aHR 0.163, 95% CI 0.091-0.290), or with 5 to 10 inpatient and outpatient hospital visits due to infectious diseases prior to 24 months old (aHR 0.058, 95% CI 0.017-0.202).

**Conclusions:**

Compared with JE-I, receiving JE-L as the most recent vaccine was associated with lower risk of inpatient and outpatient hospital visits for LRTIs among children aged 25 months to 35 months. The nature of NSEs induced by JE-L should be considered for policymakers and physicians when recommending JE vaccines to those at high risk of infection from the Japanese encephalitis virus.

## Introduction

Vaccination is widely recognized as one of the most effective measures to prevent infectious diseases and has been listed as the top priority for public health precautions by governments worldwide. According to estimates provided by the World Health Organization, 2-3 million children younger than 5 years old could be saved through vaccination against target diseases [1].

Recently, several observational studies and randomized controlled trials have suggested that, in addition to their specific effects, vaccines may have nonspecific effects (NSE) against nontargeted diseases [2]. Live vaccines such as the bacillus Calmette-Guérin (BCG) vaccine [3]; measles, mumps, and rubella (MMR) vaccine [4]; and oral polio vaccine (OPV) [5] have been observed to decrease all-cause mortality and nontargeted infectious disease hospitalizations in children, while inactivated vaccines such as the inactivated polio vaccine (IPV) [5] and diphtheria, tetanus, and pertussis (DTaP) vaccine [6] have been associated with a possible increase in all-cause mortality, on average [7,8]. Consequently, it could be extrapolated that the nature of NSEs produced by a vaccine may be associated with the type of vaccine (ie, NSEs may be more likely to be produced by a live vaccine rather than an inactivated vaccine). However, the conclusions of these previous studies are drawn from studies with a high risk of bias [6] and must be treated with caution. A high risk of bias includes selection bias and information bias arising from, for example, misclassification of vaccination status, confounding at baseline, and selective reporting (and nonreporting) of results. Currently, the immunological mechanisms underlying this phenomenon are unclear but may be related to the trained immunity generated by live vaccines, which is defined as the induction of innate immunity to generate immune memory while clearing pathogens by innate immune cells [9].

In addition, the NSEs of a live vaccine are affected by the sequence of vaccinations. Sørup et al [4] reported that receiving the inactivated DTaP-IPV-*Haemophilus influenzae* type b (Hib) vaccine as the most recent vaccine (the type of the last vaccine administered to the child) could increase the risk of hospital admissions by 62% during the first 11 months to 24 months after vaccination, while the risk decreased by 14% if MMR was the most recent vaccine. In the United States, Bardenheier et al [10] replicated the study by Sørup et al [4] and demonstrated that receiving a live vaccine as the most recent vaccine was associated with a lower risk of hospitalization for nontargeted infectious diseases from 16 months through 24 months of age compared with an inactivated vaccine (hazard ratio [HR] 0.83, 95% CI 0.72-0.94) as well as concurrent receipt compared with inactivated vaccine (HR 0.91, 95% CI 0.72-0.94).

Japanese encephalitis (JE) vaccines have been included in China’s National Immunization Program (NIP) since 2008. According to the vaccination schedule, children need be inoculated with 2 doses of the live-attenuated JE vaccine (JE-L) at 8 months and 24 months of age or with 4 doses of the inactivated JE vaccine (JE-I) at 8 months, 8 months (7-10 days after the previous dose), 24 months, and 72 months of age [11]. In China, all school-aged children who have reached the age at which they should be vaccinated are vaccinated in accordance with the current NIP vaccination procedures. The type of vaccine (attenuated or inactivated) can be chosen by the child's guardians. Otherwise, the local health authority randomly replenishes JE vaccines, and the providers administer one after another. In Hangzhou, for example, failure to receive immunization vaccines affects children's enrollment in day care and school. In contrast, children in the United States are administered only the JE-I due to safety considerations [12]. Based on the conclusions of previous studies, we hypothesized that JE-L might also have NSEs. Therefore, in this study, we aimed to evaluate whether the risk of inpatient and outpatient hospital visits for a lower respiratory tract infection (LRTI) differed among children who received the JE-L as their most recent vaccination in comparison with the JE-I.

## Methods

### Data Source and Participants

Yinzhou is an urban district of Ningbo located in the southeast coastal region of China with an estimated area of 812.40 km^2^ and 1.61 million permanent residents in 2020. A previous study [13] conducted a comprehensive surveillance of JE in Ningbo and showed that the dominant mosquito species in Ningbo was *Culex tritaeniorhynchus* and that neither JE virus nor dengue virus was detected in mosquitoes throughout the year, suggesting that the rate of local mosquito vectors carrying the virus was relatively low. In addition, another study [14,15] found the vaccine coverage against JE for children aged 1 year to 3 years in Yinzhou District in 2015 was 99.66%.

In 2005, the Yinzhou District Center for Disease Control and Prevention (CDC) developed a population-based electronic health record (EHR) database that collects information from hospitals and community health service centers in the region. This database includes general demographic characteristics, health care information, inpatient and outpatient electronic medical records, health insurance information, disease surveillance, vaccination information, management information, and death certificates [16,17]. As of 2015, it contained the health records of 1.19 million people [18].

We performed this analysis using the aforementioned database as our source of data from 2017 to 2019. Children born in Yinzhou between January 1, 2017, and December 31, 2017, who had received the second dose of JE-L or the third dose of JE-I at 24 months of age as their most recent vaccine were enrolled in this study.

### Data Collection

We collected information on demographic characteristics, vaccination (type of vaccine, vaccine name, vaccine dose, and vaccination date), inpatient and outpatient visits (visit time, admission time, discharge time), and disease diagnosis for all included participants. Data pertaining to other variables including maternal parity, birth weight, chronic diseases (including malformations of the respiratory system, other conditions associated with respiratory symptoms, neuromuscular disease, congenital diseases of the heart and urinary system, chromosomal abnormalities, and acquired chronic conditions; see Table S1 in Multimedia Appendix 1 for the specific chronic disease types and *International Statistical Classification of Diseases, Tenth Revision* [*ICD-10*] codes), and the number of inpatient and outpatient visits for infectious diseases or for all causes (see Table S2 in Multimedia Appendix 1) prior to 24 months of age were also obtained.

### Outcomes

From January 1, 2019, to December 31, 2019, we recorded the primary or secondary discharge diagnosis of LRTIs including influenza, pneumonia, pertussis, acute bronchitis, and others encoded under the diseases of the respiratory system by the *ICD-10* as the outcomes of interest (see Table S3 in Multimedia Appendix 1).

### Design

Children who had received vaccines other than JE-L or JE-I at the age of 25 months to 35 months were excluded from this study to limit the possibility of bias due to other vaccines. Further, participants with any incomplete or missing information were also excluded. The remaining participants were divided into two groups by the type of JE vaccines received at 24 months of age. Children who received JE-L as their most recent vaccine were included in the JE-L cohort, while those who received JE-I as their most recent vaccine were included in the JE-I cohort. Participants in both groups were followed from the date of administration of JE-L or JE-I to 35 months of age, death, or migration.

We calculated the incidence density of LRTIs and constructed a model to estimate the HR between the JE-L cohort and JE-I cohort.

### Statistical Analysis

We used mean (SD) or frequencies with constituent ratios to report the distribution of the children’s age, sex, chronic diseases, maternal parity, and other variables. The chi-square test and Fisher exact test were used to compare baseline characteristics between the 2 cohorts.

Since a child could have had recurring hospital visits due to an LRTI, all hospital visits needed to be involved in the analysis. We calculated the incidence density of LRTIs by using the sum of the number of inpatient and outpatient visits at 25 months to 35 months of age and divided this by the number of person-years of observation. At the same time, we assessed the average length of hospital stay by using the total number of days of hospitalization divided by the number of patients.

The Cox regression hazards model is primarily applied to a single outcome. Therefore, we decided to construct an Andersen-Gill model to assess the HRs and 95% CIs. This model is used for recurrent data and allows every participant to be presented only once and not be compared with him or herself [4]. Schoenfeld residuals were evaluated to assume the proportionality of hazards. If violations were detected, the normal Andersen-Gill model was changed to a time-dependent Andersen-Gill model. We used gender, age group, chronic diseases, birth weight, and the number of outpatient visits with or without infectious diseases prior to 24 months of age as potential covariates in the adjusted analyses. In addition, the results were stratified by sex and other variables.

Statistical analyses for this study were performed using SAS 9.4 (SAS Institute Inc). All tests were 2-sided, and *P*<.05 was considered significant.

### Sensitivity Analyses

Given that the JE-L needs to be administered in 2 doses and JE-I needs to be administered in 4 doses, sequential immunization may be undertaken in clinical practice. Meanwhile, studies have shown that the NSEs of live vaccines might be influenced by the concurrent or successive administration of inactivated vaccines. Consequently, to limit the bias caused by receiving 2 types of JE vaccine by a single child, we repeated our analyses by dividing the children into 4 groups according to the immunization course, as follows: (1) children who received JE-L only, (2) children who received JE-I after JE-L, (3) children who received JE-L after JE-I, and (4) children who received JE-I only. We calculated the incidence density of LRTIs in all 4 groups. At the same time, the Andersen-Gill model was constructed to estimate the HR for the cohort that received JE-L only compared with the cohort that received only JE-I. As per previous reports, if JE-L has NSE, the HR should be <1 [2,10].

### Ethical Considerations

The protocol for this study was approved by Zhejiang Chinese Medical University Ethics Committee (No. 20200515-1).

## Results

### Population

**Figure 1:**
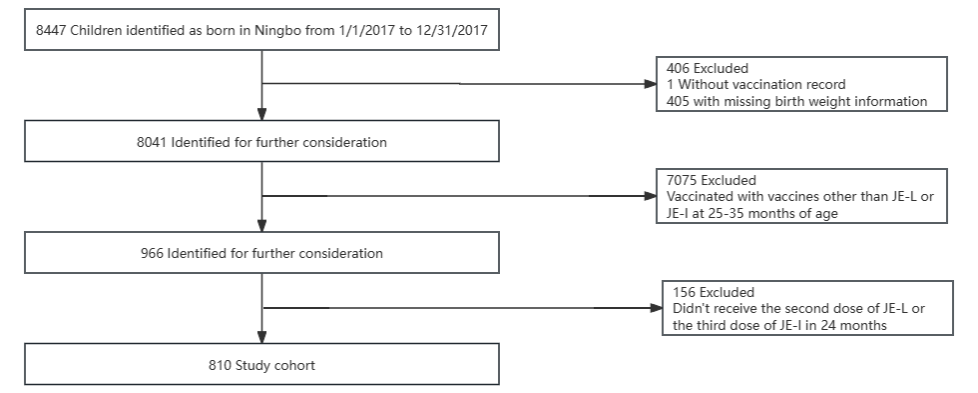
Flowchart of participant inclusion. JE-I: inactivated Japanese encephalitis vaccine; JE-L: live-attenuated Japanese encephalitis vaccine.

The study initially included 8447 children born in Yinzhou in 2017, of whom 1 child lacked vaccination records and 405 had missing weight information. Further, 7075 children who received vaccines other than those for JE at 25 months to 35 months of age were also excluded. Among the remaining children, 156 were excluded because JE was not administered as their most recent vaccine. The JE-L cohort included the 585 remaining children were administered JE-L as the most recent vaccine, and the JE-I cohort included the 225 children who were administered JE-I as the most recent vaccine, for a total of 810 participants (Figure 1).

Of the 810 children included in the final analysis, 53.1% (430/810) were male, 22.5% (182/810) had a birth weight <3000 g, 5.2% (42/810) had chronic diseases, 63.5% (514/810) had a mother with 1 parity, 30% (243/810) had visited the hospital more than 15 times prior to 24 months of age, and 6.5% (53/810) had visited the hospital more than 5 times due to infectious diseases prior to 24 months of age. Both the study cohorts were comparable in terms of sex (*P*=.81), birth weight (*P*=.25), number of hospital visits with or without infectious diseases prior to 24 months of age (*P*=.38 and *P*=.79, respectively), and chronic diseases (*P*=.41). Notably, the JE-I cohort had a higher coverage of the 13-valent pneumonia vaccine, enterovirus 71 vaccine, and rotavirus vaccine and were statistically more likely to have no siblings than the JE-L cohort (see Table S4 in Multimedia Appendix 1).

### Vaccination Status

Among the 8041 children with complete demographic and medical records in the database, 8029 children received a JE vaccine, for a coverage of 99.85%. A total of 36.26% (2916/8041) had completed the first dose of JE-I, and 63.59% (5113/8041) had completed the first dose of JE-L. The age at vaccination administration was consistent with the recommended age in the NIP. Of the 810 children included in final analysis, 27.8% (225/810) received JE-I at an average age of 24.08 months, and 72.2% (585/810) received JE-L at an average age of 24.18 months (see Table S5 in Multimedia Appendix 1).

### Inpatient and Outpatient Hospital Visits for LRTIs

Among the 810 children who received the JE vaccine as their most recent vaccine at 24 months of age, 85 reported LRTIs during the period of 25 months to 35 months of age, with 310 inpatient and outpatient hospital visits in total. The incidence density was 0.383 (95% CI 0.349-0.417) person-years. Of these 85 children, 73 had acute bronchitis, with an incidence density of 0.331 (95% CI 0.299-0.344) person-years; 8 experienced pneumonia, with an incidence density of 0.033 (95% CI 0.022-0.048) person-years; and 4 reported influenza, with an incidence density of 0.019 (95% CI 0.010-0.030) person-years. Due to LRTIs, 33 children were admitted only once, whereas 52 children were admitted ≥2 times between 25 months and 35 months of age, with a maximum of 28 admissions. The onset age was 29.25 (95% CI 28.70-29.80) months in the JE-I cohort and 29.24 (95% CI 28.78-29.69) months in the JE-L cohort, with no statistical difference (*P*=.97).

Of the 85 children with inpatient or outpatient hospital visits due to LRTIs, 5 were hospitalized for 33 days, with the shortest hospital stay being 5 days and the longest being 7 days. There were average lengths of hospitalization of 7 days in the JE-I cohort and 4.5 days in the JE-L cohort (see Table S6 in Multimedia Appendix 1).

### Risk of Inpatient and Outpatient Hospital Visits for LRTIs

In the JE-I cohort, there were 128 visits attributed to LRTIs during 225 person-years, with an incidence density of 0.568 (95% CI 0.501-0.634) person-years. In the JE-L cohort, there were 182 visits attributed to LRTIs during 585 person-years, with an incidence density of 0.311 (95% CI 0.274-0.350) person-years. Further, we performed a subgroup analysis, which revealed that the incidence density of the JE-I cohort was lower than that of the JE-L cohort (Table 1).

**Table 1:** Incidence density and hazard ratios (HRs) of inpatient and outpatient hospital visits for lower respiratory tract infections in different cohorts (N=810): live-attenuated Japanese encephalitis vaccine (JE-L; n=585), inactivated Japanese encephalitis vaccine (JE-I; n=225).

Characteristics of the cohorts				Incidence density, person-years (95% CI)		Admissions/person-years		Unadjusted HR (95% CI)		*P* value			Adjusted HR^a^ (95% CI)			*P* value	
**All characteristics**											<.001						<.001
	JE-I			0.568 (0.501-0.634)		128/225		1.000					1.000				
	JE-L			0.311 (0.274-0.350)		182/585		0.547 (0.436-0.685)					0.537 (0.416-0.693)				
**Sex**																	
	**Male**										.18						.14
		JE-I	0.529(0.436-0.620)		64/121		1.000					1.000					
		JE-L	0.430(0.374-0.488)		133/309		0.814 (0.604-1.096)					0.764 (0.534-1.093)					
	**Female**										<.001						<.001
		JE-I	0.615 (0.515-0.709)		64/104		1.000					1.000					
		JE-L	0.177 (0.134-0.228)		49/276		0.288 (0.199-0.419)					0.305 (0.198-0.469)					
**Age (months)**																	
	**25-27**										.37						.65
		JE-I	0.071 (0.020-0.173)		4/56		1.000					1.000					
		JE-L	0.119 (0.068-0.187)		15/127		1.654 (0.549-4.982)					1.384 (0.345-5.556)					
	**28-31**										.006						.34
		JE-I	0.474 (0.310-0.642)		18/38		1.000					1.000					
		JE-L	0.208 (0.148-0.282)		31/149		0.439 (0.246-0.785)					0.536 (0.150-1.908)					
	≥**32**										<.001						<.001
		JE-I	1.140 (0.943-1.362)		106/93		1.000					1.000					
		JE-L	0.504 (0.442-0.565)		136/270		0.442 (0.343-0.570)					0.454 (0.336-0.612)					
**Chronic diseases**																	
	**No**										<.001						<.001
		JE-I	0.474 (0.405-0.544)		100/211		1.000					1.000					
		JE-L	0.289 (0.252-0.329)		161/557		0.610 (0.475-0.783)					0.553 (0.420-0.729)					
	**Yes**										.001						.14
		JE-I	2.000 (1.372-2.759)		28/14		1.000					1.000					
		JE-L	0.750 (0.551-0.893)		21/28		0.375 (0.213-0.660)					0.472 (0.175-1.276)					
**Maternal parity**																	
	**1**										<.001						<.001
		JE-I	0.567 (0.486-0.646)		89/157		1.000					1.000					
		JE-L	0.244 (0.200-0.292)		87/357		0.430 (0.320-0.578)					0.361 (0.255-0.511)					
	**2**										.11						.83
		JE-I	0.574 (0.448-0.693)		39/68		1.000					1.000					
		JE-L	0.424 (0.359-0.492)		95/224		0.739 (0.509-1.073)					0.952 (0.603-1.505)					
	**3**										—^b^						—
		JE-I	0		0/0		1.000					1.000			—		
		JE-L	0		0/0		—					—			—		

^a^Andersen-Gill model adjusted for sex, birth weight, age, maternal parity, chronic diseases, number of hospital visits prior to 24 months of age, number of hospital visits prior to 24 months of age due to infectious diseases, and nonimmunization program vaccines administered before 24 months of age.

^b^Not applicable.

There was no violation detected between the 2 cohorts, as evaluated via Schoenfeld residuals to test the assumption of a proportional hazard. Therefore, we constructed a normal Andersen-Gill model to calculate the HR. The results showed the JE-L cohort had a lower risk of LRTIs than the JE-I cohort (adjusted HR [aHR] 0.537, 95% CI 0.416-0.693) in the adjusted analyses. Likewise, there were statistical differences within the subgroups (sex, age, chronic diseases, maternal parity), with a lower risk of LRTIs for female children (aHR 0.305, 95% CI 0.198-0.469), children aged 32 months to 35 months (aHR 0.454, 95% CI 0.336-0.612), children without chronic diseases (aHR 0.553, 95% CI 0.420-0.729), and children without siblings (aHR 0.361, 95% CI 0.255-0.511; Table 1).

### Types of LRTIs

In the adjusted analyses, the JE-L cohort had a lower risk of pneumonia (aHR 0.501, 95% CI 0.393-0.638) and acute bronchitis (aHR 0.525, 95% CI 0.396-0.698) than the JE-I cohort (Table 2).

**Table 2:** Incidence density and hazard ratios (HRs) of inpatient and outpatient hospital visits for different types of lower respiratory tract infection (LRTIs) in the cohorts: live-attenuated Japanese encephalitis vaccine (JE-L; n=585), inactivated Japanese encephalitis vaccine (JE-I; n=225).

Types of LRTIs by cohorts		Incidence density, person-years (95% CI)	Admissions/ person-years	Adjusted HR^a^ (95% CI)	*P* value	
**Influenza**						.39
	JE-I	0.013 (0.003-0.038)	3/225	1.000		
	JE-L	0.021 (0.011-0.036)	12/585	0.501 (0.102-2.451)		
**Pneumonia**						<.001
	JE-I	0.040 (0.018-0.075)	9/225	1.000		
	JE-L	0.031 (0.018-0.048)	18/585	0.501 (0.393-0.638)		
**Acute bronchitis**						<.001
	JE-I	0.516 (0.448-0.582)	116/225	1.000		
	JE-L	0.260 (0.225-0.297)	152/585	0.525 (0.396-0.698)		

^a^Andersen-Gill model adjusted for sex, birth weight, age, maternal parity, chronic diseases, number of hospital visits prior to 24 months of age, number of hospital visits prior to 24 months of age due to infectious diseases, and nonimmunization program vaccines administered before 24 months of age.

### Association With Timing

In the adjusted analyses, no association was observed between the time elapsed after the most recent vaccine and the occurrence of LRTIs among children aged 25 months to 35 months (Table 3).

**Table 3:** Incidence density and hazard ratios (HRs) of inpatient or outpatient hospital visits according to the time elapsed since the most recent vaccine in the cohorts: live-attenuated Japanese encephalitis vaccine (JE-L; n=585), inactivated Japanese encephalitis vaccine (JE-I; n=225).

Time after the most recent vaccine by cohort (months)		Incidence density, person-years (95% CI)	Admissions/person-years	Unadjusted HR (95% CI)	*P* value		Adjusted HR^a^ (95% CI)		*P* value	
**0-89**						.02				.13
	JE-I	5.667 (3.743-7.454)	17/3	1.000			1.000			
	JE-L	2.882 (2.215-3.626)	49/17	0.509 (0.293-0.883)			0.208 (0.027-1.581)			
**90-179**						<.001				—^b^
	JE-I	4.750 (3.151-6.388)	19/4	1.000			1.000			
	JE-L	1.538 (0.966-2.276)	20/13	0.324 (0.173-0.607)			—			
**180-269**						.09				.40
	JE-I	3.714 (2.589-4.952)	26/7	1.000			1.000			
	JE-L	2.450 (1.871-3.106)	49/20	0.659 (0.410-1.061)			0.681 (0.279-1.659)			
≥**270**						.01				.03
	JE-I	8.250 (7.238-9.009)	66/8	1.000			1.000			
	JE-L	5.333 (4.401-6.249)	64/12	0.646 (0.451-0.899)			4.295 (1.200-15.375)			

^a^Andersen-Gill model adjusted for sex, birth weight, age, maternal parity, chronic diseases, number of hospital visits prior to 24 months of age, number of hospital visits prior to 24 months of age due to infectious diseases, and nonimmunization program vaccines administered before 24 months of age.

^b^Not applicable.

### Association With the Number of Hospital Visits

In the adjusted analyses, we found that children with more hospital visits in the JE-L cohort had a lower risk of LRTIs than the children in the JE-I cohort. Prior to 24 months of age, children who visited the hospital more than 30 times (aHR 0.163, 95% CI 0.091-0.290) and those who visited the hospital 5 to 10 times owing to infectious diseases (aHR 0.058, 95% CI 0.017-0.202) had fewer hospital visits due to LRTIs (Table 4).

**Table 4:** Incidence density and hazard ratios (HRs) of inpatient and outpatient hospital visits for lower respiratory tract infections (LRTIs) according to hospital visits in the cohorts: live-attenuated Japanese encephalitis vaccine (JE-L; n=585), inactivated Japanese encephalitis vaccine (JE-I; n=225).

Number of hospital visits prior to 24 months of age by cohort				Incidence density, person-years (95% CI)		Admissions/person-years		Unadjusted HR (95% CI)		*P* value			Adjusted HR^a^ (95% CI)			*P* value	
**Any reason**																	
	**0**										—^b^						—
		JE-I	—		0/42		1.000					1.000					
		JE-L	0.042 (0.014-0.096)		5/118		—					—					
	**1-14**										.29						.40
		JE-I	0.196 (0.127-0.282)		22/112		1.000					1.000					
		JE-L	0.149 (0.111-0.195)		44/295		0.759 (0.455-1.267)					0.790 (0.459-1.361)					
	**15-29**										<.001						.003
		JE-I	0.209 (0.100-0.360)		9/43		1.000					1.000					
		JE-L	0.681 (0.587-0.766)		77/113		3.254 (1.632-6.490)					2.993 (1.448-6.185)					
	≥**30**										<.001						<.001
		JE-I	3.464 (2.908-4.053)		97/28		1.000					1.000					
		JE-L	0.949 (0.858-0.989)		56/59		0.274 (0.197-0.381)					0.163 (0.091-0.290)					
**Due to infectious causes**																	
	**0**										.93						.26
		JE-I	0.078 (0.036-0.143)		9/115		1.000					1.000					
		JE-L	0.076 (0.048-0.112)		22/291		0.966 (0.445-2.097)					0.939 (0.398-2.217)					
	**1-4**										.28						,80
		JE-I	0.347 (0.253-0.452)		33/95		1.000					1.000					
		JE-L	0.430 (0.368-0.493)		110/256		1.237 (0.838-1.825)					1.053 (0.705-1.573)					
	**5-9**										<.001						<.001
		JE-I	6.100 (5.073-7.060)		61/10		1.000					1.000					
		JE-L	1.303 (0.959-1.715)		43/33		0.214 (0.145-0.316)					0.058 (0.017-0.202)					
	≥**10**										.003						—
		JE-I	5.000 (3.553-6.447)		25/5		1.000					1.000					
		JE-L	1.400 (0.582-2.674)		7/5		0.280 (0.121-0.647)					—					

^a^Andersen-Gill model adjusted for sex, birth weight, age, maternal parity, chronic diseases, number of hospital visits prior to 24 months of age, number of hospital visits prior to 24 months of age due to infectious diseases, and nonimmunization program vaccines administered before 24 months of age.

^b^Not applicable.

### Sensitivity Analyses

During the entire course of JE immunization, 212 children received JE-I only, 573 children received JE-L only, 13 children received JE-I after JE-L, and 12 children received JE-L after JE-I. The incidence density of those who received JE-L only was lower than those who received JE-I only (0.316 vs 0.604 person-years), which is similar to the results of the main analysis (see Table S7 in Multimedia Appendix 1).

In addition, the association between the vaccination course of JE and the risk of LRTIs was also similar to that of the main analysis. Children who received JE-L only had a lower risk of inpatient and outpatient hospital visits due to LRTIs than those who received JE-I only (aHR 0.512, 95% CI 0.396-0.662), and this effect was especially pronounced in female children (aHR 0.292, 95% CI 0.189-0.449), those aged 32 months to 35 months (aHR 0.434, 95% CI 0.321-0.587), children with no siblings (aHR 0.355, 95% CI 0.251-0.502), children without chronic diseases (aHR 0.524, 95% CI 0.397-0.692), children with >30 hospital visits prior to 24 months of age (aHR 0.163, 95% CI 0.091-0.290), and children with 5 to 10 hospital visits due to infectious diseases prior to 24 months of age (aHR 0.062, 95% CI 0.017-0.218; see Table S8 in Multimedia Appendix 1).

## Discussion

### Principal Findings

Most previous studies on NSEs of vaccines have been carried out in resource-limited countries, and several have been conducted in high-income countries, such as the United States [10], Denmark [4], and The Netherlands [19]. To the best of our knowledge, this is the first study to explore the NSEs of JE vaccines in China.

This retrospective cohort study was based on information derived from an integrated clinical database to explore the NSEs of JE-L and their relevant factors. Our findings show that receiving JE-L as the most recent vaccine is associated with a lower risk of hospital admission due to LRTIs (including pneumonia and acute bronchitis) among children aged 25 months to 35 months. In addition, this study also illustrated that the NSEs of JE vaccines may be influenced by sex, health status, maternal parity, number of inpatient or outpatient hospital visits prior to 24 months of age, and the absence of siblings. There was no statistical significance between the time elapsed since the last vaccination and the onset of LRTI-related hospital admissions.

We believe that our findings are reliable since the information used in this study was acquired from Yinzhou EHR databases, which have been proved to be authentic and reliable based on our previous herpes zoster study and other studies [16,18,20-23]. In addition, we had access to the complete immunization records of the children included in this study (vaccination information was obtained from the Immunization Administration Registry and the CDC Adverse Event Following Immunization Information System).

Among other studies, vaccines currently shown to have NSEs include BCG, MMR, measles, live polio, and DPT. In China, these vaccines are all NIP vaccines, which can be given to children free of charge. Some studies have reported that the vaccine coverage of NIP vaccines in China is consistently above 80% [24], and in Yinzhou, the coverage rate is even higher (96.22%) [14]. Almost all children were vaccinated. Therefore, the NSEs effect found in this study cannot be attributed to BCG, MMR, and other related vaccines.

Consistent with the findings of previous studies, our study showed that the incidence density of LRTIs at 25 months to 35 months of age among the 810 participants was much higher among male children and those with a mother with more than 1 parity. Unlike other countries, China's one-child policy, which was in place for nearly 40 years (1979-2015), fundamentally altered the country's demographic and social structure. The fact that most of our study participants were a single child is also influenced by the change in policy [25]. One study reported that the risk of hospital admissions for children with respiratory syncytial virus was related to maternal parity and gender of the children in the family and that children with siblings (adjusted odds ratio [OR] 1.96) and male children (adjusted OR 1.57) were at higher risk [26]. A meta-analysis also identified male sex (OR 1.23) and having siblings (OR 1.60) as risk factors for developing LRTIs [27]. Thus, having 1 child may protect against secondary LRTIs from siblings. Meanwhile, we also found that the incidence density of LRTIs was higher in children with chronic diseases and those of older age, which is in agreement with the findings by Sørup et al [4].

Several reports have shown that administration of live vaccines such as BCG [3,6,28], MMR [4,19,29], and OPV [5,30,31] as the most recent vaccine may have NSEs that decrease all-cause mortality or the risk of hospital admissions due to nontarget infectious diseases. In their study, Sørup et al [4] found that receiving MMR as the most recent vaccine, compared with DTaP-IPV-Hib, was associated with a 20% (95% CI 16%-24%) lower risk of LRTIs. In addition, Sørup et al [5] also found that OPV could reduce the risk of LRTIs by 15% (95% CI 5%-23%) compared with DTaP-IPV-Hib as the most recent vaccine. Hollm-Delgado et al [32] found that the BCG vaccine was associated with 17% to 35% risk reduction of LRTIs. A systematic review [2] also suggested that live vaccines could lower the risk of developing LRTIs. Furthermore, Bardenheier et al [10] reported that live vaccines could reduce hospitalizations due to nontarget LRTIs by 44% to 64%. Therefore, our results indicating that JE-L has NSEs on LRTIs are consistent with those of the aforementioned reports.

Although the possible biological mechanisms to support our findings have not been identified, trained immunity [9,33] can be assumed to play a major role. It is now believed that trained immunity is the result of the interaction between immunity, metabolism, and epigenetics. Alterations in the metabolism of intrinsic immune cells affect epigenetics, which can further influence metabolic pathways and cytokine production [34]. For example, the epigenetic modifications in the body after BCG vaccination led to an increase of H3K4me3 at the promoter regions of certain genes, producing protection against unrelated pathogens [35]. Further, it can be conjectured that heterologous protective immunity (cross-protection via T cells) might have a minor role in producing the NSEs of vaccines. In addition, compared with JE-I, JE-L has been reported to produce much more interferon-γ spot-forming cells and interleukin-2 spot-forming cells, both of which exert important antiviral effects [36].

Studies have also found that the NSEs of live vaccines are sex-specific to some extent and that the NSEs of BCG [37,38] and the measles vaccine [39,40] favor the female sex, whereas the NSEs of OPV [30,41] favor the male sex in all-cause mortality and hospital admissions. These results are similar to our findings showing that the NSEs of JE-L favors female children. This observation may be related to the fact that female children are more likely to have a stronger and faster innate and adaptive immune respoNSEs to viruses than male children due to the higher number, activity, and inflammatory immune respoNSEs of innate immune cells, including monocytes, macrophages, and dendritic cells in female children than male children [42-44]. The other possible reasons might be the hormone-mediated pro-inflammatory effects of low-dose estradiol and anti-inflammatory effects of testosterone and progesterone in those of the female sex [2,45,46].

Notably, our results did not elicit protective effects of JE-L on those with chronic diseases, which may be related to the limited number of children with chronic diseases included in the study. Regarding the association between the NSEs of JE-L and the number of hospital visits, our study showed that more hospital visits had a greater protective effect of JE-L in children. This was in agreement with the results of the study by Sørup et al [4], which reported that the NSEs of MMR were better in children with a higher frequency of hospital admissions for infectious diseases before the age of 11 months (0 vs 1 vs 2 vs 3 times and above: HR 1.00 vs 0.96 vs 0.92 vs 0.86, respectively) and children who had been admitted before 11 months of age (0 vs 1 times and above: HR 1.00 vs 0.80). Meanwhile, similar to our findings, the study by Sørup et al [4] also reported that the NSEs of MMR are better in children with mothers with 1 parity than with mothers with 2 parities (HR 1.00 vs 1.03).

Moreover, we also performed a sensitivity analysis in our study to ensure the reliability of our results. The sensitivity analysis in this study revealed similar results to the main analysis, which further supported the finding that JE-L has NSEs on LRTIs. Additionally, it verified that the NSEs of JE-L could be diminished by JE-I.

### Limitations

This study had several limitations. First, the limited number of participants who met the inclusion criteria resulted in a wide confidence interval for the results, which may have reduced the reliability of our study. Second, we cannot exclude some underreporting, which may bias the results toward uncorrelation. Third, due to the limitations of the database, our study failed to include potential confounders that may have influenced the results. For example, studies have demonstrated that parental smoking [4] and socioeconomic status [47] can influence hospital admissions and the highest educational level of the child’s guardian can influence vaccination choices [48]. Consequently, more studies on the NSEs of JE-L based on EHR databases with sufficient variables are needed in the future. Fourth, the low number of cases of LRTIs found in this study may be related to the vaccination of children against pneumonia [14] and influenza [49]. This may mask the NSEs of JE vaccines.

It is noteworthy that most countries recommend JE-I rather than JE-L for those with an immunocompromised status, such as patients with rheumatic diseases [50], HIV, or solid organ transplant [51,52] owing to possible safety concerns (ie, JE-L was contraindicated in immunocompromised individuals in Australia) [53]. However, inoculating healthy children with JE-L is recommended for its stronger protection [54,55] and may be associated with fewer side effects than JE-I [56,57]. According to our data, the nature of NSEs induced by JE-L should be considered by policymakers and physicians when recommending JE vaccines to those at high risk of infection from the JE virus.

Finally, as this study was conducted just prior to the COVID-19 epidemic, it would be valuable to repeat the study after COVID-19 to validate the robustness of the results in further studies, as COVID-19–related isolation and societal changes may have influenced susceptibility to LRTIs in the study cohort.

### Conclusions

Compared with JE-I, receiving JE-L as the most recent vaccine was associated with a lower risk of hospital visits for LRTIs among children aged 25 months to 35 months.

## Appendix

Multimedia Appendix 1 Supplementary tables.

## Abbreviations

aHR: adjusted hazard ratio
BCG: bacillus Calmette-Guérin
CDC: Center for Disease Control and Prevention
DTaP: diphtheria, tetanus, and pertussis
EHR: electronic health record
Hib: *Haemophilus influenzae* type b
HR: hazard ratio
*ICD-10*: *International Statistical Classification of Diseases, Tenth Revision*
IPV: inactivated polio vaccine
JE: Japanese encephalitis
JE-I: inactivated Japanese encephalitis vaccine
JE-L: live-attenuated Japanese encephalitis vaccine
LRTI: lower respiratory tract infection
MMR: measles, mumps, and rubella
NIP: National Immunization Program
NSE: nonspecific effects
OPV: oral polio vaccineOR: odds ratio

## Data Availability

The data that support the findings of this study are available from the Yinzhou Center for Disease Prevention and Control (CDC). Restrictions apply to the available of these data, which were used under license for this study. Data are available with permission from the Yinzhou CDC.

## References

[1] Koppaka R, Global Public Health Achievements Team (2011). Ten great public health achievements --- worldwide, 2001--2010. Morbidity and Mortality Weekly Report (MMWR).

[2] de Bree L, Koeken VA, Joosten LA, Aaby P, Benn CS, van Crevel R, Netea MG (2018). Non-specific effects of vaccines: Current evidence and potential implications. Semin Immunol.

[3] Biering-Sørensen S, Aaby P, Lund N, Monteiro I, Jensen KJ, Eriksen HB, Schaltz-Buchholzer F, Jørgensen ASP, Rodrigues A, Fisker AB, Benn CS (2017). Early BCG-Denmark and neonatal mortality among infants weighing less than 2500 g: a randomized controlled trial. Clin Infect Dis.

[4] Sørup S, Benn CS, Poulsen A, Krause TG, Aaby P, Ravn H (2014). Live vaccine against measles, mumps, and rubella and the risk of hospital admissions for nontargeted infections. JAMA.

[5] Sørup S, Stensballe LG, Krause TG, Aaby P, Benn CS, Ravn H (2016). Oral polio vaccination and hospital admissions with non-polio infections in Denmark: nationwide retrospective cohort study. Open Forum Infect Dis.

[6] Higgins JPT, Soares-Weiser K, López-López JA, Kakourou A, Chaplin K, Christensen H, Martin NK, Sterne JAC, Reingold AL (2016). Association of BCG, DTP, and measles containing vaccines with childhood mortality: systematic review. BMJ.

[7] Aaby P, Garly ML, Nielsen J, Ravn H, Martins C, Balé C, Rodrigues A, Benn CS, Lisse IM (2007). Increased female-male mortality ratio associated with inactivated polio and diphtheria-tetanus-pertussis vaccines: Observations from vaccination trials in Guinea-Bissau. Pediatr Infect Dis J.

[8] Aaby P, Benn C, Nielsen J, Lisse IM, Rodrigues A, Ravn H (2012). Testing the hypothesis that diphtheria-tetanus-pertussis vaccine has negative non-specific and sex-differential effects on child survival in high-mortality countries. BMJ Open.

[9] Gyssens I, Netea M (2019). Heterologous effects of vaccination and trained immunity. Clin Microbiol Infect.

[10] Bardenheier BH, McNeil MM, Wodi AP, McNicholl JM, DeStefano F (2017). Risk of nontargeted infectious disease hospitalizations among US children following inactivated and live vaccines, 2005-2014. Clin Infect Dis.

[11] (2021). National Immunization Program Vaccine Childhood Immunization Schedule (2021 Edition)EB/OL 05/16. Chinese Center for Disease Control and Prevention.

[12] (2023). Japanese Encephalitis ACIP Vaccine Recommendations. Centers for Disease Control and Prevention.

[13] Xiao M, Chaoyang H, Yinjun L (2013). Analysis of the comprehensive monitoring results of epidemic Japanese encephalitis in Ningbo City. Chinese Journal of Vector Biology and Control.

[14] Yexiang S, Hongbo L, Yin M (2017). Monitoring and evaluation of vaccination rates for 1-3 year old children in Yinzhou District, Ningbo City, Zhejiang Province in 2015 J. Chinese Journal of Vaccines and Immunization.

[15] Hu Y, Chen Y (2017). Evaluating childhood vaccination coverage of NIP vaccines: coverage survey versus Zhejiang provincial immunization information system. Int J Environ Res Public Health.

[16] Wang J, Bao B, Shen P, Kong G, Yang Y, Sun X, Ding G, Gao B, Yang C, Zhao M, Lin H, Zhang L (2019). Using electronic health record data to establish a chronic kidney disease surveillance system in China: protocol for the China Kidney Disease Network (CK-NET)-Yinzhou Study. BMJ Open.

[17] Lu F, Xu C, Zhang P, Xu Y, Liu J (2021). Construction and implementation of big data in healthcare in Yichang City, Hubei Province. China CDC Wkly.

[18] Yang Y, Zhou X, Gao S, Lin H, Xie Y, Feng Y, Huang K, Zhan S (2018). Evaluation of electronic healthcare databases for post-marketing drug safety surveillance and pharmacoepidemiology in China. Drug Saf.

[19] Tielemans SMAJ, de Melker HE, Hahné SJM, Boef AGC, van der Klis FRM, Sanders EAM, van der Sande MAB, Knol MJ (2017). Non-specific effects of measles, mumps, and rubella (MMR) vaccination in high income setting: population based cohort study in the Netherlands. BMJ.

[20] Huang K, Tao S, Zhou X, Mo J, Zhu B, Shen P, Lin H, Arena PJ, He N (2020). Incidence rates of health outcomes of interest among Chinese children exposed to selected vaccines in Yinzhou Electronic Health Records: A population-based retrospective cohort study. Vaccine.

[21] Sun X, Wei Z, Lin H, Jit M, Li Z, Fu C (2021). Incidence and disease burden of herpes zoster in the population aged ≥50 years in China: Data from an integrated health care network. J Infect.

[22] Zhang D, Tang X, Shen P, Si Y, Liu X, Xu Z, Wu J, Zhang J, Lu P, Lin H, Gao P (2019). Multimorbidity of cardiometabolic diseases: prevalence and risk for mortality from one million Chinese adults in a longitudinal cohort study. BMJ Open.

[23] li H, lin H, Zhao H, Xu Y, Cheng Y, Shen P, Zhan S (2018). Statins use and risk of new-onset diabetes in hypertensive patients: a population-based retrospective cohort study in Yinzhou district, Ningbo city, People's Republic of China. TCRM.

[24] Jia Y, Lei C, Wen Y, Yi S, Zun Y (2022). Routine immunization reporting rate for National Immunization Program vaccines, China, 2020-2021. Chinese Journal of Vaccines and Immunization.

[25] Cai Y, Feng W (2021). The social and sociological consequences of China's one-child policy. Annu. Rev. Sociol.

[26] Paynter S, Ware RS, Lucero MG, Tallo V, Nohynek H, Weinstein P, Williams G, Sly PD, Simões EAF (2014). Malnutrition: a risk factor for severe respiratory syncytial virus infection and hospitalization. Pediatr Infect Dis J.

[27] Shi T, Balsells E, Wastnedge E, Singleton R, Rasmussen ZA, Zar HJ, Rath BA, Madhi SA, Campbell S, Vaccari LC, Bulkow LR, Thomas ED, Barnett W, Hoppe C, Campbell H, Nair H (2015). Risk factors for respiratory syncytial virus associated with acute lower respiratory infection in children under five years: Systematic review and meta-analysis. J Glob Health.

[28] Roth A, Jensen H, Garly M, Djana Q, Martins CL, Sodemann M, Rodrigues A, Aaby P (2004). Low birth weight infants and Calmette-Guérin bacillus vaccination at birth: community study from Guinea-Bissau. Pediatr Infect Dis J.

[29] La Torre G, Saulle R, Unim B, Meggiolaro A, Barbato A, Mannocci A, Spadea A (2017). The effectiveness of measles-mumps-rubella (MMR) vaccination in the prevention of pediatric hospitalizations for targeted and untargeted infections: A retrospective cohort study. Hum Vaccin Immunother.

[30] Lund N, Andersen A, Hansen ASK, Jepsen FS, Barbosa A, Biering-Sørensen S, Rodrigues A, Ravn H, Aaby P, Benn CS (2015). The effect of oral polio vaccine at birth on infant mortality: a randomized trial. Clin Infect Dis.

[31] Seppälä E, Viskari H, Hoppu S, Honkanen H, Huhtala H, Simell O, Ilonen J, Knip M, Hyöty H (2011). Viral interference induced by live attenuated virus vaccine (OPV) can prevent otitis media. Vaccine.

[32] Hollm-Delgado MG, Stuart EA, Black RE (2014). Acute lower respiratory infection among Bacille Calmette-Guérin (BCG)-vaccinated children. Pediatrics.

[33] Messina N, Zimmermann P, Curtis N (2019). The impact of vaccines on heterologous adaptive immunity. Clin Microbiol Infect.

[34] Butkeviciute E, Jones CE, Smith SG (2018). Heterologous effects of infant BCG vaccination: potential mechanisms of immunity. Future Microbiol.

[35] Covián C, Fernández-Fierro A, Retamal-Díaz A, Díaz FE, Vasquez AE, Lay MK, Riedel CA, González PA, Bueno SM, Kalergis AM (2019). BCG-induced cross-protection and development of trained immunity: implication for vaccine design. Front Immunol.

[36] Li M, Yu Y, Liu X (2010). Comparative study on the cellular immunity response induced by live attenuated SA14-14-2 Japanese encephalitis vaccine and inactivated Japanese encephalitis vaccine in mice. Chinese Journal of Vaccines and Immunization.

[37] Stensballe LG, Nante E, Jensen IP, Kofoed P, Poulsen A, Jensen H, Newport M, Marchant A, Aaby P (2005). Acute lower respiratory tract infections and respiratory syncytial virus in infants in Guinea-Bissau: a beneficial effect of BCG vaccination for girls community based case-control study. Vaccine.

[38] Roth A, Sodemann M, Jensen H, Poulsen A, Gustafson P, Weise C, Gomes J, Djana Q, Jakobsen M, Garly M, Rodrigues A, Aaby P (2006). Tuberculin reaction, BCG scar, and lower female mortality. Epidemiology.

[39] Aaby P, Samb B, Simondon F, Knudsen K, Seck AM, Bennett J, Markowitz L, Rhodes P, Whittle H (1994). Sex-specific differences in mortality after high-titre measles immunization in rural Senegal. Bull World Health Organ.

[40] Aaby P, Jensen H, Samb B, Cisse B, Sodemann M, Jakobsen M, Poulsen A, Rodrigues A, Lisse IM, Simondon F, Whittle H (2003). Differences in female-male mortality after high-titre measles vaccine and association with subsequent vaccination with diphtheria-tetanus-pertussis and inactivated poliovirus: reanalysis of West African studies. The Lancet.

[41] Upfill-Brown A, Taniuchi M, Platts-Mills JA, Kirkpatrick B, Burgess SL, Oberste MS, Weldon W, Houpt E, Haque R, Zaman K, Petri WA (2017). Nonspecific effects of oral polio vaccine on diarrheal burden and etiology among Bangladeshi infants. Clin Infect Dis.

[42] Klein SL (2012). Sex influences immune responses to viruses, and efficacy of prophylaxis and treatments for viral diseases. Bioessays.

[43] Ruggieri A, Anticoli S, D'Ambrosio A, Giordani L, Viora M (2016). The influence of sex and gender on immunity, infection and vaccination. Ann Ist Super Sanita.

[44] Klein SL, Marriott I, Fish EN (2015). Sex-based differences in immune function and responses to vaccination. Trans R Soc Trop Med Hyg.

[45] Fischinger S, Boudreau CM, Butler AL, Streeck H, Alter G (2019). Sex differences in vaccine-induced humoral immunity. Semin Immunopathol.

[46] de Araújo Albuquerque LP, da Silva AM, de Araújo Batista FM, de Souza Sene I, Costa DL, Costa CHN (2021). Influence of sex hormones on the immune response to leishmaniasis. Parasite Immunol.

[47] Bettenhausen JL, Colvin JD, Berry JG, Puls HT, Markham JL, Plencner LM, Krager MK, Johnson MB, Queen MA, Walker JM, Latta GM, Riss RR, Hall M (2017). Association of income inequality with pediatric hospitalizations for ambulatory care-sensitive conditions. JAMA Pediatr.

[48] Wu J, Wei Z, Yang Y, Sun X, Zhan S, Jiang Q, Fu C (2023). Gap between cognitions and behaviors among children's guardians of influenza vaccination: The role of social influence and vaccine-related knowledge. Hum Vaccin Immunother.

[49] Lixia Y, Ting F, Rui M (2019). Analysis of seasonal influenza vaccination rates among children aged 6-35 months in Ningbo, Zhejiang Province from 2010 to 2018. Chinese Journal of Vaccines and Immunization.

[50] Welzel T, Wörner A, Heininger U (2020). [Travel vaccinations in rheumatic diseases : Specific considerations in children and adults]. Z Rheumatol.

[51] Aung A, Trubiano J, Spelman D (2015). Travel risk assessment, advice and vaccinations in immunocompromised travellers (HIV, solid organ transplant and haematopoeitic stem cell transplant recipients): A review. Travel Med Infect Dis.

[52] Chang L, Lim BCW, Flaherty GT, Torresi J (2019). Travel vaccination recommendations and infection risk in HIV-positive travellers. J Travel Med.

[53] Kleinnijenhuis J, Quintin J, Preijers F, Joosten LAB, Ifrim DC, Saeed S, Jacobs C, van Loenhout J, de Jong D, Stunnenberg HG, Xavier RJ, van der Meer JWM, van Crevel R, Netea MG (2012). Bacille Calmette-Guerin induces NOD2-dependent nonspecific protection from reinfection via epigenetic reprogramming of monocytes. Proc Natl Acad Sci U S A.

[54] Furuya-Kanamori L, Xu C, Doi SAR, Clark J, Wangdi K, Mills DJ, Lau CL (2021). Comparison of immunogenicity and safety of licensed Japanese encephalitis vaccines: A systematic review and network meta-analysis. Vaccine.

[55] Hegde NR, Gore MM (2017). Japanese encephalitis vaccines: Immunogenicity, protective efficacy, effectiveness, and impact on the burden of disease. Hum Vaccin Immunother.

[56] WHO (2016). Japanese Encephalitis Vaccines: WHO position paper, February 2015--Recommendations. Vaccine.

[57] Islam N, Lau C, Leeb A, Mills D, Furuya-Kanamori L (2022). Safety profile comparison of chimeric live attenuated and Vero cell-derived inactivated Japanese encephalitis vaccines through an active surveillance system in Australia. Hum Vaccin Immunother.

