# Supplementary material for: Investigating Nonspecific Effects of the Live-Attenuated Japanese Encephalitis Vaccine on Lower Respiratory Tract Infections in Children Aged 25-35 Months: Retrospective Cohort Study
Source: JMIR Public Health Surveill. 2024 Mar 18;10:e53040. doi: 10.2196/53040 (PMC10993859; doi:10.2196/53040)
Supplement: Multimedia Appendix 1 [file publichealth_v10i1e53040_app1.docx]

Table S1. Overview of the ICD-10 Codes Used to Define Chronic Conditions of Children.

|  | **Diagnoses** | ***ICD10–codes*** |
| --- | --- | --- |
| Malformations of the respiratory system | Cleft lip and palate | Q35.0–Q37.9 |
|  | Malformations of the larynx, trachea, and bronchi | Q31.0–Q31.9 |
|  |  | Q32.0–Q32.4 |
|  | Malformations of the lungs | Q33.0–Q33.9 |
|  | Other malformations of the respiratory system | Q30.0–Q30.9 |
|  |  | Q34.0–Q34.9 |
| Other conditions associated with respiratory symptoms | Bronchopulmonary dysplasia | P27.0–P27.9 |
|  | Cystic fibrosis | E84.0–E84.9 |
|  | Esophageal atresia | Q39.0–Q39.1 |
|  | Other malformations of the esophagus | Q39.2–Q39.9 |
|  | Congenital diaphragmatic hernia | Q79.0 |
| Neuromuscular disease | Encephalocele | Q01.0–Q01.9 |
|  | Microcephalus | Q02.9 |
|  | Congenital hydrocephalus | Q03.0–Q03.9 |
|  | Other cerebral malformations | Q04.0–Q04.9 |
|  | Other malformations in the nervous system | Q07.0–Q07.9 |
|  | Spina bifida and malformations of the spinal cord | Q05.0–Q05.9 |
|  |  | Q06.0–Q06.9 |
|  | Spinal muscular atrophy | G12.0–G12.9 |
|  | Muscular dystrophy | G71.0–G71.3 |
|  | Congenital disturbances of muscle tonus, peripheral nerve disease, congenital myasthenia | P94.1–P94.9 |
|  |  | G60.0–G60.9 |
|  |  | G70.2 |
|  | Cerebral palsy | G80.0–G80.9 |
| Congenital diseases of the heart and the urinary system, chromosomal abnormalities, and others | Congenital heart disease | Q20.0–Q26.9 |
|  |  | I27.0 |
|  | Malformations of the urinary system | N13.0–N13.9 |
|  |  | Q60.0–Q64.9 |
|  | Conditions affecting renal function | N07.0–N07.9 |
|  |  | N25.0–N25.9 |
|  | Down syndrome | Q90.0–Q90.9 |
|  | Other chromosomal abnormalities | Q91.0–Q99.9 |
|  | Malformations of the gastrointestinal tract, liver, biliary system, pancreas, and the abdominal wall | Q40.0–Q43.9 |
|  |  | Q44.0–Q44.7 |
|  |  | Q45.0–Q45.3 |
|  |  | Q79.2–Q79.3 |
|  | Congenital immunodeficiencies | D80.0–D82.9 |
|  | Inborn errors of metabolism | E70.0–E73.0 |
|  |  | E74.0–E83.9 |

Table S1. Overview of the ICD-10 Codes Used to Define Chronic Conditions of Children (Continued).

|  | **Diagnoses** | ***ICD10–codes*** |
| --- | --- | --- |
| Congenital diseases of the heart and the urinary system, chromosomal abnormalities, and others | Congenital viral infection | P35.0–P35.9 |
|  | Fetal alcohol syndrome | Q86.0 |
| Acquired Chronic Conditions | Interstitial lung disease | J84.0–J84.9 |
|  | Gastroesophageal reflux | K21.0–K21.9 |
|  | Epilepsy | G40.0–G40.9 |
|  | Hydrocephalus | G91.0–G91.9 |
|  | Acquired heart disease | I27.1–I27.9 |
|  |  | I30.0–I52.8 |
|  | Conditions affecting renal function | N03.0–N03.9 |
|  |  | N05.0–N05.9 |
|  |  | N18.0–N19.9 |
|  |  | N26.0–N27.9 |
|  | Cancer | C00.0–C80.9 |
|  |  | C81.0–C96.9 |
|  | Liver disease | K70.0–K77.8 |
|  | Malabsorption | K90.0–K90.9 |
|  | Autoimmune disease | M30.0–M35.9 |

Table S2. Overview of the ICD-10 Codes Used to Define Admission Due to Infections.

|  | **Diagnoses** | ***ICD10–codes*** |
| --- | --- | --- |
| Upper respiratory  infections | Abscessus peritonsillaris | J36.0–J36.9 |
|  | Infections in the ear | H65.0–H67.9; H68.0; H70.0–H70.9 |
|  | Laryngitis | J37.0; A36.2; J04.0–J05.9 |
|  | Nasopharyngitis | A36.1; J00.0–J00.9 |
|  | Pharyngitis | J02.0–J02.9 |
|  | Sinuitis | J32.0–J32.9; J01.0–J01.1; J01.3–J01.9 |
|  | Ethmoiditis | J01.2 |
|  | Tonsillitis | J35.0; A36.0; J03.0–J03.9 |
|  | Other | A36.8–A36.9; J06.0–J06.9 |
| Lower respiratory  infections | Influenza | J09.0–J11.9 |
|  | Pneumonia | A48.1; A70.0–A70.9; J12.0–J18.9 |
|  | Other acute lower respiratory  infections | A37.0–A37.9; A42.0; J20.0-J22.9; J85.0–J86.9 |
| Gastrointestinal  infections |  | A00.0–A02.0; A02.2–A09.9; A42.1 |
| Other infections | Acute lymphadenitis | L04.0–L04.9 |
|  | Cellulitis and abscess | H60.0–H60.1; L02.0–L03.9 |
|  | Dermatophytosis and other superficial mycoses | B35.0–B36.9 |
|  | Erysipelas | A46.0–A46.9 |
|  | Viral warts | B07.0–B07.9 |
|  | Other local infections of skin and subcutaneous tissue | A36.3; H60.2–H60.4; H60.8–H60.9; L00.0–L01.1; L08.0–L08.9; L30.3 |
|  | Certain bacterial diseases | A20.0–A32.0; A32.8–A35.9; A38.0–A38.9; A39.1; A39.3–A39.9; A42.2–A44.9; A48.0; A48.2–A49.9; A50.0–A53.9; A54.0–A54.9; A55.0–A56.9; A57.0–A57.9; A74.8–A74.9 |
|  | Cystitis | N30.0 |
|  | Hepatitis | B15.0–B19.9 |
|  | Infections of the circulatory system | I00.0–I01.9; I30.0–I30.9; I33.0–I33.9; I38.0–I38.9; I39.8–I40.9 |
|  | Infections of the eye | A71.0–A74.0; H00.0–H01.0; H01.8–H01.9; H03.0–H03.1; H06.1; H10.0; H10.2–H10.3; H10.5–H10.8 |
|  | Infections of the musculoskeletal system and connective tissue | M00.0–M01.9; M60.0; M86.3–M86.6 |

Table S2. Overview of the ICD-10 Codes Used to Define Admission Due to Infections (Continued).

|  | **Diagnoses** | ***ICD10–codes*** |
| --- | --- | --- |
|  | Infections of the nervous system | G04.0–G07.9 |
|  | Infections of the urinary system | N34.0–N34.1; N34.3 |
|  | Meningitis | A32.1; A39.0; A87.0–A87.9; B00.3–B00.4; B01.0–B01.1; B02.0–B02.1; B05.0–B05.1; B06.0; B26.1–B26.2; G00.0–G03.9 |
|  | Mycoses | B37.0–B37.6; B37.8–B49.9 |
|  | Nephritis | N08.0 |
|  | Osteomyelitis | M46.2; M46.5; M86.0–M86.2 |
|  | Protozoal diseases, helminthiases, pediculosis, acariasis and other infestations | A59.0–A59.9; B50.0–B89.9 |
|  | Pyelonephritis | N10.9 |
|  | Rickettsiosis | A75.0–A79.9 |
|  | Sepsis | A02.1; A32.7; A39.2; A40.0–A41.9;  B37.7 |
|  | Spirochaetal disease | A65.0–A69.9 |
|  | Tuberculosis | A15.0–A19.9; K93.0 |
|  | Unspecified infections | B99.0–B99.9 |
|  | Viral infections | A58.0–A58.9; A60.0–A60.9; A63.0; A80.0–A86.9; A88.0–A99.9; B00.0–B00.2; B00.5–B00.9; B01.2–B01.9; B02.2–B04.9; B05.2–B05.9; B06.1–B06.9; B08.0–B09.9; B20.0–B26.0; B26.3–B34.9 |
|  | Others | A63.8–A64.9 |

Table S3. Overview of the ICD-10 Codes Used to Define Admission Due to Lower Respiratory Tract Infections.

|  | **Diagnoses** | ***ICD10–codes*** |
| --- | --- | --- |
| Lower respiratory  infections | Influenza | J09.0–J11.9 |
|  | Pneumonia | A48.1; A70.0–A70.9; J12.0–J18.9 |
|  | Other acute lower respiratory  infections | A37.0–A37.9; A42.0; J20.0-J22.9; J85.0–J86.9 |

Table S4. Descriptive Characteristics of The Children in Yinzhou District, Ningbo City.

|  | **Characteristic** | **Total** | |  | **The JE-I cohort** | |  | **The JE-L cohort** | | ***χ^2^*** | ***P-value*** |
| --- | --- | --- | --- | --- | --- | --- | --- | --- | --- | --- | --- |
|  |  | **N** | **%** |  | **N** | **%** |  | **N** | **%** |  |  |
| Sex | Male | 430 | 53.09 |  | 121 | 53.78 |  | 309 | 52.82 | 0.0598 | .807 |
|  | Female | 380 | 46.91 |  | 104 | 46.22 |  | 276 | 47.18 |  |  |
| Parity | 1 | 514 | 63.46 |  |  |  |  | 357 | 61.03 | NA | .037 |
|  | 2 | 292 | 36.05 |  | 157 | 69.78 |  | 224 | 38.29 |  |  |
|  | 3 | 4 | 0.49 |  | 68 | 30.22 |  | 4 | 0.68 |  |  |
| Birth weight (g) | <2000 | 9 | 1.11 |  | NA | NA |  | 9 | 1.54 | 4.116 | .249 |
|  | 2000- | 173 | 21.36 |  | 46 | 20.44 |  | 127 | 21.71 |  |  |
|  | 3000- | 575 | 70.98 |  | 162 | 72.00 |  | 413 | 70.60 |  |  |
|  | 4000- | 53 | 6.54 |  | 17 | 7.56 |  | 36 | 6.15 |  |  |
| Number of hospital visits prior to 24 months of age | 0 | 160 | 19.75 |  | 42 | 18.67 |  | 118 | 20.17 | 1.0453 | .790 |
|  | 1- | 407 | 50.25 |  | 112 | 49.78 |  | 295 | 50.43 |  |  |
|  | 15- | 156 | 19.26 |  | 43 | 19.11 |  | 113 | 19.32 |  |  |
|  | 30- | 87 | 10.74 |  | 28 | 12.44 |  | 59 | 10.09 |  |  |
| Number of hospital visits prior to 24 months of age due to infectious diseases | 0 | 406 | 50.12 |  | 115 | 51.11 |  | 291 | 49.75 | 3.0492 | .384 |
|  | 1- | 351 | 43.33 |  | 95 | 42.22 |  | 224 | 43.76 |  |  |
|  | 5- | 43 | 5.31 |  | 10 | 4.44 |  | 55 | 5.64 |  |  |
|  | 10- | 10 | 1.23 |  | 5 | 2.22 |  | 7 | 0.85 |  |  |
| Chronic disease | No | 768 | 94.81 |  | 211 | 93.78 |  | 557 | 95.21 | 0.6815 | .409 |
|  | Yes | 42 | 5.19 |  | 14 | 6.22 |  | 28 | 4.79 |  |  |
| 13-valent pneumonia vaccine | No | 659 | 81.36 |  | 143 | 63.56 |  | 516 | 88.21 | NA | <0.001 |
|  | Yes | 151 | 18.64 |  | 82 | 36.44 |  | 69 | 11.79 |  |  |
| Varicella vaccine | No | 10 | 1.23 |  | NA | NA |  | 10 | 1.71 | NA | .070 |
|  | Yes | 800 | 98.77 |  | 225 | 100 |  | 575 | 98.29 |  |  |
| Influenza vaccine | No | 493 | 60.86 |  | 137 | 60.89 |  | 356 | 60.85 | 0.0001 | .993 |
|  | Yes | 317 | 39.14 |  | 88 | 39.11 |  | 229 | 39.15 |  |  |
| EV-71 vaccine | No | 439 | 54.20 |  | 105 | 46.67 |  | 334 | 57.09 | 7.1176 | .008 |
|  | Yes | 371 | 45.80 |  | 120 | 53.33 |  | 251 | 42.91 |  |  |
| Rotavirus vaccine | No | 649 | 80.12 |  | 168 | 74.67 |  | 481 | 82.22 | 5.8249 | .016 |
|  | Yes | 161 | 19.88 |  | 57 | 25.33 |  | 104 | 17.78 |  |  |

Table S5. The Vaccination Status of Children.

|  | **JE vaccine** | **N** | **%** | **Vaccination age** |
| --- | --- | --- | --- | --- |
| 8041 children | JE-I |  |  |  |
|  | The first dose | 2916 | 36.26 | 8.49 |
|  | The second dose | 2912 | 36.21 | 8.86 |
|  | The third dose | 2950 | 36.69 | 24.10 |
|  | JE-L |  |  |  |
|  | The first dose | 5113 | 63.59 | 8.39 |
|  | The second dose | 4825 | 60.00 | 24.14 |
| 810 children | JE-I | 225 | 27.78 | 24.08 |
|  | JE-L | 585 | 72.22 | 24.18 |

Table S6. The Hospitalization of the Children with LRTI.

|  | **Total** | **The JE-I cohort** | **The JE-L cohort** |
| --- | --- | --- | --- |
| The length of hospitalization (days) |  |  |  |
| Max | 7.0 | 7.0 | 7.0 |
| Min | 5.0 | 7.0 | 5.0 |
| In average | 6.6 | 7.0 | 6.5 |
| The number of hospitalized children | 5 | 1 | 4 |

Table S7. The Incidence Density of In- and Out-hospital Visits for LRTI in Different Cohorts according to the immunization course of JE Vaccine.

|  | **The vaccination of JE vaccine** | **Admissions/ Person-years** | **Incidence density (95%*CI*)** |
| --- | --- | --- | --- |
| All children |  |  |  |
|  | Received JE-I only | 128/212 | 0.604(0.535-0.670) |
|  | Received JE-I after JE-L | 0/13 | NA |
|  | Received JE-L after JE-I | 1/12 | 0.083(0.002-0.385) |
|  | Received JE-L only | 181/573 | 0.316(0.278-0.356) |
| Sex |  |  |  |
| Male | Received JE-I only | 64/114 | 0.561(0.465-0.654) |
|  | Received JE-I after JE-L | 0/7 | NA |
|  | Received JE-L after JE-I | 1/8 | 0.125(0.003-0.527) |
|  | Received JE-L only | 132/301 | 0.439(0.382-0.497) |
| Female | Received JE-I only | 64/98 | 0.653(0.550-0.764) |
|  | Received JE-I after JE-L | 0/6 | NA |
|  | Received JE-L after JE-I | 0/4 | NA |
|  | Received JE-L only | 49/272 | 0.180(0.136-0.231) |
| Age |  |  |  |
| 25- | Received JE-I only | 4/55 | 0.073(0.020-0.176) |
|  | Received JE-I after JE-L | 0/1 | NA |
|  | Received JE-L after JE-I | 1/6 | 0.167(0.004-0.641) |
|  | Received JE-L only | 14/121 | 0.116(0.065-0.187) |
| 28- | Received JE-I only | 18/31 | 0.581(0.391-0.755) |
|  | Received JE-I after JE-L | 0/7 | NA |
|  | Received JE-L after JE-I | 0/1 | NA |
|  | Received JE-L only | 31/148 | 0.209(0.147-0.284) |
| 32- | Received JE-I only | 106/88 | 1.205(0.997-1.438) |
|  | Received JE-I after JE-L | 0/5 | NA |
|  | Received JE-L after JE-I | 0/4 | NA |
|  | Received JE-L only | 136/266 | 0.511(0.449-0.573) |
| Chronic diseases |  |  |  |
| No | Received JE-I only | 100/198 | 0.505(0.433-0.577) |
|  | Received JE-I after JE-L | 0/13 | NA |
|  | Received JE-L after JE-I | 1/12 | 0.083(0.002-0.385) |
|  | Received JE-L only | 160/545 | 0.293(0.256-0.334) |
| Yes | Received JE-I only | 28/14 | 2.000(1.372-2.759) |
|  | Received JE-I after JE-L | 0/0 | NA |
|  | Received JE-L after JE-I | 0/0 | NA |
|  | Received JE-L only | 21/28 | 0.750(0.551-0.893) |
| Parity |  |  |  |
| 1 | Received JE-I only | 89/149 | 0.597(0.514 -0.677) |
|  | Received JE-I after JE-L | 0/8 | NA |
|  | Received JE-L after JE-I | 1/8 | 0.125(0.003-0.527) |
|  | Received JE-L only | 86/349 | 0.246(0.202-0.295) |
| 2 | Received JE-I only | 39/63 | 0.619(0.488-0.739) |
|  | Received JE-I after JE-L | 0/5 | NA |
|  | Received JE-L after JE-I | 0/4 | NA |
|  | Received JE-L only | 95/220 | 0.432(0.365-0.500) |
| 3 | Received JE-I only | NA | NA |
|  | Received JE-I after JE-L | NA | NA |
|  | Received JE-L after JE-I | NA | NA |
|  | Received JE-L only | 0/4 | NA |

Table S8. The Hazard Ratios of In- and Out-hospital visits for LRTI in Different Cohorts according to the immunization course of JE Vaccine.

|  | **The vaccination of JE vaccine** | **Adjusted *HR^a^* (95%*CI*)** | ***P-value*** |
| --- | --- | --- | --- |
| All children | Received JE-I only | 1.000 | <.001 |
|  | Received JE-L only | 0.512 (0.396-0.662) |  |
| Sex |  |  |  |
| Male | Received JE-I only | 1.000 | .063 |
|  | Received JE-L only | 0.708 (0.492-1.019) |  |
| Female | Received JE-I only | 1.000 | <.001 |
|  | Received JE-L only | 0.292 (0.189-0.449) |  |
| Chronic diseases |  |  |  |
| No | Received JE-I only | 1.000 | <.001 |
|  | Received JE-L only | 0.524 (0.397-0.692) |  |
| Yes | Received JE-I only | 1.000 | .139 |
|  | Received JE-L only | 0.472 (0.175-1.276) |  |
| Age (months) |  |  |  |
| 25- | Received JE-I only | 1.000 | .690 |
|  | Received JE-L only | 1.038 (0.239-4.508) |  |
| 28- | Received JE-I only | 1.000 | .244 |
|  | Received JE-L only | 0.468 (0.130-1.680) |  |
| 32- | Received JE-I only | 1.000 | <.001 |
|  | Received JE-L only | 0.434 (0.321-0.587) |  |
| Parity |  |  |  |
| 1 | Received JE-I only | 1.000 | <.001 |
|  | Received JE-L only | 0.355 (0.251-0.502) |  |
| 2 | Received JE-I only | 1.000 | .541 |
|  | Received JE-L only | 0.867 (0.548-1.371) |  |
| 3 | Received JE-I only | 1.000 | NA |
|  | Received JE-L only | NA |  |
| The number of hospital visit prior 24 months |  |  |  |
| 0- | Received JE-I only | 1.000 | NA |
|  | Received JE-L only | NA |  |
| 1- | Received JE-I only | 1.000 | .154 |
|  | Received JE-L only | 0.670 (0.387-1.162) |  |
| 15- | Received JE-I only | 1.000 | .005 |
|  | Received JE-L only | 2.847 (1.375-5.893) |  |
| 30 | Received JE-I only | 1.000 | <.001 |
|  | Received JE-L only | 0.163 (0.091-0.290) |  |
| The number of hospital visit prior 24 months due to infectious |  |  |  |
| 0 | Received JE-I only | 1.000 | .907 |
|  | Received JE-L only | 0.950 (0.403-2.242) |  |
| 1- | Received JE-I only | 1.000 | .894 |
|  | Received JE-L only | 0.973 (0.650-1.456) |  |
| 5- | Received JE-I only | 1.000 | <0.001 |
|  | Received JE-L only | 0.062 (0.017-0.218) |  |
| 10- | Received JE-I only | 1.000 | NA |
|  | Received JE-L only | NA |  |
| ^a^ AG model adjusted for sex, birth weight, age, parity, chronic diseases, number of hospital visits prior to 24 months of age, number of hospital visits prior to 24 months of age due to infectious diseases, and non-immunization program vaccines administered before 24 months of age. | | | |
